# Model-Based Spike Detection of Epileptic EEG Data

**DOI:** 10.3390/s130912536

**Published:** 2013-09-17

**Authors:** Yung-Chun Liu, Chou-Ching K. Lin, Jing-Jane Tsai, Yung-Nien Sun

**Affiliations:** 1 Department of Computer Science and Information Engineering, National Cheng Kung University, No. 1, University Road, Tainan City 701, Taiwan; E-Mail: yungchun7@gmail.com; 2 Medical Device Innovation Center, National Cheng Kung University, No. 1, University Road, Tainan City 701, Taiwan; 3 Department of Neurology, National Cheng Kung University Hospital, No. 138, Sheng Li Road, Tainan City 704, Taiwan; E-Mails: cxl45@mail.ncku.edu.tw (C.-C.K.L.); epitsai@mail.ncku.edu.tw (J.-J.T.)

**Keywords:** epilepsy, slow wave, spike detection, spike classification, nonlinear energy operator

## Abstract

Accurate automatic spike detection is highly beneficial to clinical assessment of epileptic electroencephalogram (EEG) data. In this paper, a new two-stage approach is proposed for epileptic spike detection. First, the k-point nonlinear energy operator (k-NEO) is adopted to detect all possible spike candidates, then a newly proposed spike model with slow wave features is applied to these candidates for spike classification. Experimental results show that the proposed system, using the AdaBoost classifier, outperforms the conventional method in both two- and three-class EEG pattern classification problems. The proposed system not only achieves better accuracy for spike detection, but also provides new ability to differentiate between spikes and spikes with slow waves. Though spikes with slow waves occur frequently in epileptic EEGs, they are not used in conventional spike detection. Identifying spikes with slow waves allows the proposed system to have better capability for assisting clinical neurologists in routine EEG examinations and epileptic diagnosis.

## 1. Introduction

Epilepsy is a common brain disease [1]. To monitor the functional disorders of the brain, the most popular way is to measure the electroencephalogram (EEG), which is a measurement of the electrical potentials produced by the brain [2]. Diagnosis of epilepsy is usually based on the presence of typical epileptiform patterns, such as spikes and sharp waves, in the EEG [1]. Visual scanning of EEG recordings for these patterns remains the most common approach, though it is very laborious and time-consuming [3–5]. Furthermore, disagreement among neurologists concerning the same data may occur due to subjective differences [6]. Therefore, to alleviate the drawbacks caused by subjective manual inspection, automatic detection of epileptiform patterns, based on objective criteria, would be of great benefit to clinical diagnosis and quantitative analysis.

Various automatic spike detection algorithms have been previously published [3,4]. Algorithms are generally categorized into the following methods: template matching [7], mimetic analysis [8,9], power spectral analysis [9,10], wavelet analysis [11] and artificial neural networks (ANNs) [5,12,13]. Features, obtained from mimetic, power spectral or wavelet analysis, are usually treated as inputs to ANNs. Many conventional methods adopting different classifiers together with single spike related features for spike classification were reported in [5,8,14,15]. In clinical EEG data, it is often observed that a spike is followed by a slow wave [1]. Both spikes and spikes with slow waves are regarded as positive epileptiform patterns by neurologists. Several papers [16–18] have mentioned the existence of slow waves following the spike, but the slow wave was not utilized in the spike detection. In 2011, Ji's group [7] proposed to use some features of slow waves in their spike detection algorithms. They used slow wave features (amplitude and duration) directly in some threshold rules to help in defining spike candidates and decreasing the false positives. Their experiments supported the fact that the slow waves did help in the detection of epileptic spikes.

The proposed system adopts a two-stage approach to reduce the computational load of spike classification. The k-point nonlinear energy operator (k-NEO) is utilized first to detect all possible spike candidates, and then the proposed spike model is applied only to these candidates for further classification. The proposed spike model is designed to include both the conventional single spike model and ones with slow wave-related morphological features. Adaptive Boost (AdaBoost) classifiers were trained and used to classify EEG patterns into three classes including single spike, spike with slow wave and non-spike. The experiments demonstrate that the proposed system can successfully handle not only the two-class classification (*i.e.*, classifying the EEG signal into spike and non-spike patterns), but also the three-class classification (*i.e.*, classifying the EEG signal into spike, spike with slow wave and non-spike classes). Moreover, the proposed slow wave features are proven effective in improving spike classification. The system provides higher detection sensitivity and classification accuracy in clinical applications and is more consistent with expert neurological assessments. Details of the proposed system are provided in the sections that follow.

## 2. Materials

Twelve scalp EEG datasets from different epileptic patients and three from normal subjects were used in this study. These datasets were anonimized and randomly selected from the EEG data bank of National Cheng Kung University Hospital, Taiwan, by neurologists J. J. Tasi and C. C. K. Lin. The EEG sensors were attached according to the international 10–20 electrode system and EEGs were recorded by a Medelec-Profile system (Oxford Instruments, Old Woking, UK). EEG data was filtered by a 1–70 Hz band-pass filter and sampled at a rate of 256 Hz. Sixteen-channel EEGs (locations Fp1, Fp2, F3, F4, F7, F8, T3, T4, T5, T6, C3, C4, P3, P4, O1 and O2) from the recorded data were reviewed by the neurologists who identified 126 trials containing epileptiform patterns. Because neurologists usually review 10-s EEG recordings per page in clinical diagnosis from the PC screen, the length of each trial was set to 10 s. Within the 126 trials, the total number of epileptiform patterns confidently identified by the neurologists included 42 single spikes and 100 spikes with slow waves. The numbers of single spikes and spikes with slow waves for each EEG dataset are listed in Table 1. These data were used in the experiments to verify the proposed processes, including candidate detection and training and testing of AdaBoost classifiers.

## 3. Methods

**Figure 1.**
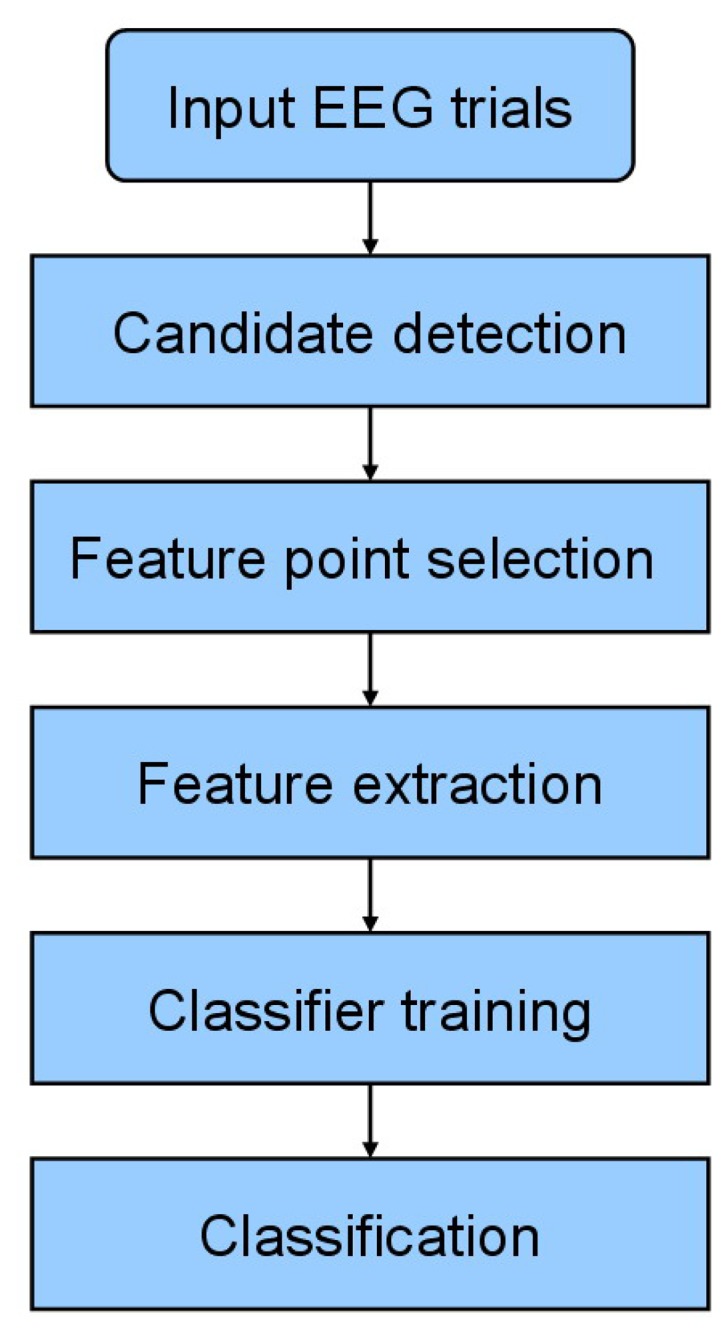
Flowchart of the proposed system.

The proposed system utilizes a two–stage approach for spike detection. Since an epileptic spike consists of two types of patterns (*i.e.*, single spikes and spikes with slow waves), we adopt k-NEO to detect all possible spike candidates which are then classified by the AdaBoost classifier using the features of the newly proposed spike model. Figure 1 shows the flowchart of the proposed method. The candidate detection, feature point selection, feature extraction and classification procedures are detailed below.

### 3.1. Candidate Detection

Before candidate detection, each EEG trial is normalized by the following two steps: the mean value and standard deviation of each trial, respectively denoted as trial_avg_ and trial_std_, are calculated. Then, each point of the EEG trial is subtracted by trial_avg_ and then divided by trial_std_.

NEO is good at detecting the sudden occurrence of high frequency signals and is commonly used in signal processing, image processing and AM/FM demodulation [19,20]. NEO is shown in Equation (1), with *x*(*n*) denoting the input sample. However, it is also sensitive to high noisy peaks, especially in low SNR situations. Choi [21] proposed the extended version k-NEO, in Equation (2), where *k* is a resolution parameter and related to the peak width of the detected spike candidates. Chatrian [1] stated that a typical spike is transient with a pointed peak and a duration of 20 to 70 ms. Thus *k* should be set to an integer between 2.56 to 8.96 (*i.e.*, an integer from 3 to 8) for a sampling rate of 256 Hz. The higher the *Ψ_k_*, the higher possibility the spike candidate is detected. Parameter *k* is empirically selected to be 3 in this study. A Hamming window of length 4*k* + 1 is adopted to eliminate noise after k-NEO processing [21]. After normalization and smoothing, a pre-defined threshold T is used to detect possible spike candidates. If a time point has a *Ψ_k_* value greater than T and also an upward peak in the normalized data, then it is regarded as a spike pattern candidate:

(1) $$\psi \{x(n)\}=x^{2}(n)-x(n-1)x(n+1)$$

(2) $$\psi_{k}\{x(n)\}=x^{2}(n)-x(n-k)x(n+k)$$

### 3.2. Feature Point Selection for the Proposed Model

**Figure 2.**
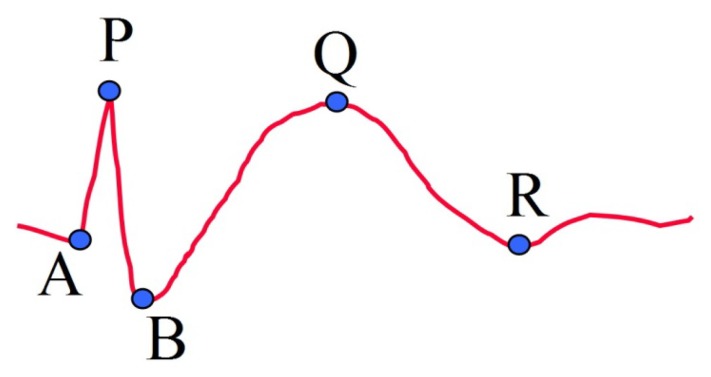
Feature points on the proposed model.

For a candidate point P, detected by k-NEO, we define four other important feature points of the proposed model (Figure 2). The group of points A, P and B represent the portion of spike, while the group of points B, Q and R represent the slow wave portion. A single spike has only the first group of points, while a spike followed by a slow wave has both two groups of points. The first two feature points are denoted as A and B, which correspond to two points on the left and right hand sides of the spike around peak point P, respectively. For point A, we trace backwards from point P until a point with a positive slope appears. Point B is selected similarly in the forward direction.

After the spike portion is processed, the reference points for the slow wave portion are selected. The two remaining feature points Q and R correspond to the local maximum and the end point of a slow wave, respectively. Firstly, the EEG signal is low-pass filtered with a cut-off frequency at 5 Hz. Tracing the EEG signal forward, point Q is the highest point after point P and point R is the lowest point after point Q. The value of cut-off frequency for lowpass filtering was determined by sequentially trying integer frequencies between 1 and 10 against the EEG data used in this study.

### 3.3. Feature Extraction

**Figure 3.**
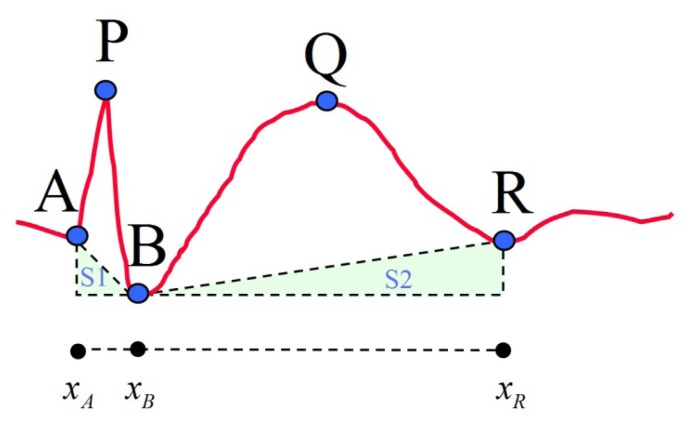
Reference diagram for feature extraction.

After the five feature points of the proposed model are selected, 13 features are then calculated for subsequent classification. These 13 features are divided into four categories, including duration, amplitude, slope and area. The calculations and descriptions of these features are listed in Table 2 and a reference diagram for feature extraction is given (Figure 3). The conventional spike model for spike pattern detection in Acir's research [5] is formed by six features: Dur_AP, Dur_PB, Amp_AP, Amp_PB, Slope_AP and Slope_PB. This feature set is denoted as FS1. The proposed spike model is designed to include both the conventional spike features and the slow wave related features (Dur_slowwave, Amp_slowwave and Area_slowwave). Collectively, these features are denoted as FS2. In addition, FS3 is also investigated in this study. It contains the nine features in FS2 and four additional spike related features of Dur_spike, Amp_spike, Slope_sharpness and Area_spike. The above-mentioned feature sets are then used in the classification module to compare spike classification performance. The three selected feature sets are outlined in Table 3.

### 3.4. Classification

AdaBoost [22] is a popular machine learning algorithm [23]. The algorithm generates a strong classifier by integrating a set of weak classifiers. These weak classifiers are trained and recruited sequentially in a series of T rounds (T = 100 in this study). At each round, AdaBoost computes the weighted classification error using the following equation:

(3) $${ɛ}_{t}=\overset{N}{\underset{i=1}{\text{∑}}}p_{i}^{t}I(h_{t}(x_{i})\neq y_{i})$$

where *x_i_* is a vector for observation *i*, *y_i_* is the class label for *x_i_*, and *h_t_* is the prediction at round *t*. Indicator function *I* is set to 1 if the predicate of *I* holds else set to 0. Classification error *ε_t_* is calculated and used in weight updating process. Weight $p_{i}^{t}$ of observation *i* at round *t* is decreased if its corresponding classification is correct and increased otherwise. Thus, the new weak classifier at round *t* + 1 may enhance the identification of difficult observations and increase system classification capability. After training, AdaBoost computes the label assignment for new observation *x* using the following equation:

(4) $$h_{f}(x)=\arg\max_{y\in Y}\overset{T}{\underset{t=1}{\text{∑}}}\left(\log\frac{1}{\beta_{t}})I(h_{t}(x)=y_{i}\right)$$

where *β_t_* = *ε_t_*/(1 − *ε_t_*).

From the 126 trials, a total of 253 spike candidates were acquired by k-NEO. Among the candidates, 42 were classified as single spikes, 100 candidates as spikes with slow waves, and the 111 remaining candidates were regarded as non-spike patterns. The experiments were used to evaluate the performance of spike classification which discriminates the EEG patterns into spike and non-spike classes. For the two-class classification, the single spike and spike with slow wave datasets were combined together to form the spike class dataset. Three feature sets (in Table 3) of spike and non-spike data were then used to train AdaBoost for classification. Similarly, for evaluating the performance of three-class classification, the feature sets of the three classes are used to train AdaBoost for classifying EEG patterns into spike, spike with slow wave and non-spike classes. The experiments are described in detail in Sections 4.2 and 4.3.

## 4. Results and Discussion

### 4.1. Candidate Detection

In the following experiments, each 10 second EEG trial contained many signal fluctuations where only 1 or 2 spike-like short duration patterns (in tens of milliseconds) could be detected. The remaining EEG fluctuations were regarded as background signals in spike detection. All 42 single spikes and 100 spikes with slow waves were correctly detected as candidates in the experimental data by k-NEO without any miss-detections. However, the number of detected candidates is related to the selected threshold T from the candidate detection procedure. The lower the threshold T, the more spike candidates would be acquired resulting in more computation in the subsequent processing. On the other hand, the higher the threshold T, the less the number of candidates would be selected and the higher the chance to harbor false negatives. Threshold T was set to 1.8 empirically in all our experiments.

### 4.2. Two-Class Classification

This section evaluates the performance of spike classification. Based on the confusion matrix [24], TP, FP, TN and FN denote the number of true positives, false positives, true negatives and false negatives, respectively. The accuracy of the classification was calculated by the following equation:

(5) $$\text{Accuracy}=\frac{TP+TN}{TP+FP+TN+FN}$$

If the samples are not large enough or are unevenly distributed in the training and testing datasets, system performance might be negatively affected. In order to reduce this effect, a four-fold cross-validation process was employed. The four-fold was selected based on the available number of trials. Too large a fold number which makes each fold have too small a number of trials will make the results less statistically meaningful. The experimental dataset was randomly divided into four groups. Each of the four groups alternately served as the testing dataset with the other three groups combined to be training dataset. The testing results from the four groups are summed up to obtain the testing statistics of a four-fold test instance. This four-fold test process was repeated ten times to obtain the final statistics for each classification experiment.

Statistical sensitivity and specificity were computed for the performance of the classification system. Sensitivity reflected the ability of detecting spikes, while specificity evaluated the ability of discriminating non-spikes.

The quantities were defined as:

(6) $$\text{Sensitivity}=\frac{TP}{TP+FN},$$

and:

(7) $$\text{Specificity}=\frac{TN}{TN+FP}.$$

For the two-class classification experiments, 142 candidate patterns including single spikes and spikes with slow waves constituted the spike class, and the remaining 111 were the non-spike class. AdaBoost was trained using 100 weak classifiers, each consisting of a single decision tree, then tested via the four-fold cross-validation procedure. System performance of the different feature sets is shown in Table 4. Although the training performance with the conventional spike model (FS1) reached 99.3%, the corresponding testing performance decreased to 87.4%. Using the proposed spike model (FS2), the result for training reached 100% and testing performance reached 93.9%. In comparison with FS1, this significant improvement of 6.5% shows that the proposed spike model was significantly more accurate in spike classification. Performance of the FS3 feature set showed similar results to FS2 in the testing phase, which implies the additional features are not more effective in spike classification. These additional features did not provide additional value to the model most likely because the FS3 exclusive features were composites or combinations of existing features and therefore did not contain any new information. Sensitivity and specificity of the system is shown in Table 5 and gives similar results to Table 4. Using the proposed spike model, sensitivity and specificity achieved significant improvements by 7.6% and 5.7%, respectively.

### 4.3. Three-Class Classification

For the three-class classification experiments, 42 patterns constituted the single spike class, 100 patterns comprised the class of spike with slow wave, and the remaining 111 were the non-spike class. AdaBoost, using 100 weak classifiers for classifying the three pattern classes, was also trained and tested via four-fold cross-validation. The accuracy of classification was calculated by dividing the total number of correct classifications by the number of total employed patterns. System performance obtained by the four-fold cross-validation process is shown in Table 6. The classification results using the FS1 feature set obtained the worst performance. The conventional spike model could not identify some of the spikes with slow waves. However, the proposed spike model (FS2) showed significantly better results than FS1, and applying FS3 for three-class classification did not yield better performance. As spikes with slow waves are common in clinical EEGs, the newly proposed spike model is more realistic for clinical spike analysis.

A pseudo-two-class classification can be derived from the results of three-class classification by combining the corresponding values for the spike and spike with slow wave classification results. The performance of the pseudo-two-class classification is shown in Tables 7 and 8. Comparing the pseudo-two-class results to the original two-class classification results reveals a slightly lower training dataset accuracy, especially for FS1. The accuracy results of the test dataset between the two-class and pseudo-two-class data are comparable for all three feature sets. This implies the training for the two-class classifier is more effective than the three-class classifier because of less complex decision boundaries. In comparing the classification accuracy of FS2 to FS1 for pseudo-two-class, there was significant improvement of 7.4% and 4.9% for both training and testing datasets, respectively. However, the classification results of FS2 and FS3 for pseudo-two-class are very similar, analogous to the relationship of FS2 and FS3 in the two-class classification results. The results for FS1 and FS2 in Tables 6 and 7 are identical, because there were no misclassification between spikes and spikes with slow waves in the three-class classification. Naturally, the three-class classification provides the additional new information by identifying spikes with slow waves in epileptic EEG diagnosis.

### 4.4. Parameter Setting of T in Candidate Detection

For clinical applications, it is important to know if the fixed threshold T is also effective for healthy subjects. Thus, EEG data from three normal subjects (N1, N2 and N3), recorded in the same way as described in the Materials section, were included. One hundred and twenty trials (40 from each subject) were selected. The results showed that 13 spike candidates, three from N1, six from N2, and four from N3, were detected with T set to 1.8. The 13 detected candidates were then classified by the AdaBoost classifiers with FS2 feature set. All 13 candidates were classified into the non-spike class. Thus, the threshold value was also good for all 120 trials from healthy subjects.

## 5. Conclusions

In this paper, a new two–stage approach was proposed for epileptic EEG spike detection. At the first stage, k-NEO is utilized to detect all possible spike candidates. At the second stage, a newly proposed spike model is then used to classify the candidate patterns into spike and non-spike classes. Since the classification load is small and k-NEO is computationally fast, the proposed approach is very efficient for spike detection. The proposed spike model augments the pattern of spikes with slow waves which are common in clinical EEGs for the conventional model. The four-fold cross-validation process with ten repetitions was employed in all experiments. Our experimental results showed that the accuracy of spike detection can be greatly improved (6.5% and 21.5% in accuracy for two- and three-class classification) by adding the slow wave model in spike detection. Thus, the proposed system reflects potential for clinical applications in EEG epileptic diagnosis. However, the classification performance and parametric setting, e.g., *k* and T, are usually affected by the adopted training samples. As we have only limited number of patient data in the current experimental setup, we will recruit more cases for system improvement and clinical validation in the future.

## Figures and Tables

**Table 1.** The numbers of single spikes and spikes with slow waves for each EEG dataset.

**EEG Dataset No.**	**Number of Single Spikes**	**Number of Spikes with Slow Waves**
1	0	16
2	0	14
3	0	7
4	0	18
5	8	9
6	0	17
7	1	2
8	30	0
9	1	2
10	0	10
11	0	3
12	2	2

Total	42	100

**Table 2.** Thirteen features analyzed in this study.

**Feature Name**	**Calculation**	**Description**
Dur_AP	${\bar{AP}}_{x}$	Duration of 1st half-wave of spike; projection of $\vec{AP}$ onto x-axis
Dur_PB	${\bar{PB}}_{x}$	Duration of 2nd half-wave of spike; projection of $\vec{PB}$ onto x-axis
Dur_spike	${\bar{AP}}_{x}+{\bar{PB}}_{x}$	Sum of durations of 1st and 2nd half-waves of spike
Dur_slowwave	${\bar{BQ}}_{x}+{\bar{QR}}_{x}$	Sum of durations of 1st and 2nd half-waves of slow wave
Amp_AP	${\bar{AP}}_{y}$	Amplitude of 1st half-wave of spike; projection of $\vec{AP}$ onto y-axis
Amp_PB	${\bar{PB}}_{y}$	Amplitude of 2nd half-wave of spike; projection of $\vec{PB}$ onto y-axis
Amp_spike	$({\bar{AP}}_{y}+{\bar{PB}}_{y})/2$	Average of amplitudes of 1st and 2nd half-waves of spike
Amp_slowwave	$({\bar{BQ}}_{y}+{\bar{QR}}_{y})/2$	Average of amplitudes of 1st and 2nd half-waves of slow wave
Slope_AP	${\bar{AP}}_{y}/{\bar{AP}}_{x}$	Slope of 1st half-wave of spike
Slope_PB	$-{\bar{PB}}_{y}/{\bar{PB}}_{x}$	Slope of 2nd half-wave of spike
Slope_sharpness	Slope_AP-Slope_PB	Sharpness of spike
Area_spike	$\int_{x_{A}}^{x_{B}}f(x)dx-S_{1}$	Area of spike (Note *f*(*x*) denotes the EEG data flow)
Area_slowwave	$\int_{x_{B}}^{x_{R}}f(x)dx-S_{2}$	Area of slow wave

**Table 3.** Feature sets for different models.

**Feature Set**	**Features**	**Number of Features**	**Notes**
FS1	Dur_AP, Dur_PB, Amp_AP, Amp_PB, Slope_AP and Slope_PB	6	Conventional spike model
FS2	FS1, Dur_slowwave, Amp_slowwave and Area_slowwave	9	The proposed spike model
FS3	FS2, Dur_spike, Amp_spike, Slope_sharpness and Area_spike	13	The proposed spike model with four additional spike related features

**Table 4.** System performance of the two-class classification using different feature sets.

**Feature Set**	**Accuracy_Train (%)**	**Accuracy_Test (%)**
FS1	99.3 ± 0.8	87.4 ± 3.2
FS2	100.0 ± 0.0	93.9 ± 2.4
FS3	100.0 ± 0.0	93.5 ± 2.6

**Table 5.** Sensitivity and specificity of the two-class classification using different feature sets.

**Feature Set**	**Sensitivity_Test (%)**	**Specificity_Test (%)**
FS1	87.9 ± 4.7	86.7 ± 5.3
FS2	95.5 ± 3.4	92.4 ± 4.7
FS3	94.4 ± 3.6	92.3 ± 4.8

**Table 6.** System performance of the three-class classification using different feature sets.

**Feature Set**	**Accuracy_Train (%)**	**Accuracy_Test (%)**
FS1	72.3 ± 1.5	70.9 ± 2.7
FS2	96.3 ± 1.5	92.4 ± 3.1
FS3	96.2 ± 1.7	92.2 ± 4.1

**Table 7.** System performance of the pseudo-two-class classification results.

**Feature Set**	**Accuracy_Train (%)**	**Accuracy_Test (%)**
FS1	88.9 ± 1.5	87.5 ± 2.9
FS2	96.3 ± 1.5	92.4 ± 3.1
FS3	96.2 ± 1.7	92.2 ± 4.1

**Table 8.** Sensitivity and specificity of the pseudo-two-class classification results.

**Feature Set**	**Sensitivity_Test (%)**	**Specificity_Test (%)**
FS1	85.5 ± 5.1	90.0 ± 5.3
FS2	94.6 ± 4.1	89.6 ± 6.8
FS3	94.4 ± 4.1	89.5 ± 7.4
